# Measurement of the $\eta _c(1S)$ production cross-section in proton–proton collisions via the decay ${\eta _c(1S)\,{\rightarrow } \,{{{ p}}} \overline{{{{ p}}}}}$

**DOI:** 10.1140/epjc/s10052-015-3502-x

**Published:** 2015-07-04

**Authors:** R. Aaij, C. Abellán Beteta, B. Adeva, M. Adinolfi, A. Affolder, Z. Ajaltouni, S. Akar, J. Albrecht, F. Alessio, M. Alexander, S. Ali, G. Alkhazov, P. Alvarez Cartelle, A. A. Alves, S. Amato, S. Amerio, Y. Amhis, L. An, L. Anderlini, J. Anderson, R. Andreassen, M. Andreotti, J. E. Andrews, R. B. Appleby, O. Aquines Gutierrez, F. Archilli, A. Artamonov, M. Artuso, E. Aslanides, G. Auriemma, M. Baalouch, S. Bachmann, J. J. Back, A. Badalov, C. Baesso, W. Baldini, R. J. Barlow, C. Barschel, S. Barsuk, W. Barter, V. Batozskaya, V. Battista, A. Bay, L. Beaucourt, J. Beddow, F. Bedeschi, I. Bediaga, S. Belogurov, K. Belous, I. Belyaev, E. Ben-Haim, G. Bencivenni, S. Benson, J. Benton, A. Berezhnoy, R. Bernet, M.-O. Bettler, M. van Beuzekom, A. Bien, S. Bifani, T. Bird, A. Bizzeti, P. M. Bjørnstad, T. Blake, F. Blanc, J. Blouw, S. Blusk, V. Bocci, A. Bondar, N. Bondar, W. Bonivento, S. Borghi, A. Borgia, M. Borsato, T. J. V. Bowcock, E. Bowen, C. Bozzi, T. Brambach, J. Bressieux, D. Brett, M. Britsch, T. Britton, J. Brodzicka, N. H. Brook, H. Brown, A. Bursche, G. Busetto, J. Buytaert, S. Cadeddu, R. Calabrese, M. Calvi, M. Calvo Gomez, P. Campana, D. Campora Perez, A. Carbone, G. Carboni, R. Cardinale, A. Cardini, L. Carson, K. Carvalho Akiba, G. Casse, L. Cassina, L. Castillo Garcia, M. Cattaneo, Ch. Cauet, R. Cenci, M. Charles, Ph. Charpentier, M. Chefdeville, S. Chen, S.-F. Cheung, N. Chiapolini, M. Chrzaszcz, K. Ciba, X. Cid Vidal, G. Ciezarek, P. E. L. Clarke, M. Clemencic, H. V. Cliff, J. Closier, V. Coco, J. Cogan, E. Cogneras, V. Cogoni, L. Cojocariu, P. Collins, A. Comerma-Montells, A. Contu, A. Cook, M. Coombes, S. Coquereau, G. Corti, M. Corvo, I. Counts, B. Couturier, G. A. Cowan, D. C. Craik, M. Cruz Torres, S. Cunliffe, R. Currie, C. D’Ambrosio, J. Dalseno, P. David, P. N. Y. David, A. Davis, K. De Bruyn, S. De Capua, M. De Cian, J. M. De Miranda, L. De Paula, W. De Silva, P. De Simone, D. Decamp, M. Deckenhoff, L. Del Buono, N. Déléage, D. Derkach, O. Deschamps, F. Dettori, A. Di Canto, H. Dijkstra, S. Donleavy, F. Dordei, M. Dorigo, A. Dosil Suárez, D. Dossett, A. Dovbnya, K. Dreimanis, G. Dujany, F. Dupertuis, P. Durante, R. Dzhelyadin, A. Dziurda, A. Dzyuba, S. Easo, U. Egede, V. Egorychev, S. Eidelman, S. Eisenhardt, U. Eitschberger, R. Ekelhof, L. Eklund, I. El Rifai, E. Elena, Ch. Elsasser, S. Ely, S. Esen, H.-M. Evans, T. Evans, A. Falabella, C. Färber, C. Farinelli, N. Farley, S. Farry, RF Fay, D. Ferguson, V. Fernandez Albor, F. Ferreira Rodrigues, M. Ferro-Luzzi, S. Filippov, M. Fiore, M. Fiorini, M. Firlej, C. Fitzpatrick, T. Fiutowski, P. Fol, M. Fontana, F. Fontanelli, R. Forty, O. Francisco, M. Frank, C. Frei, M. Frosini, J. Fu, E. Furfaro, A. Gallas Torreira, D. Galli, S. Gallorini, S. Gambetta, M. Gandelman, P. Gandini, Y. Gao, J. García Pardiñas, J. Garofoli, J. Garra Tico, L. Garrido, C. Gaspar, R. Gauld, L. Gavardi, G. Gavrilov, A. Geraci, E. Gersabeck, M. Gersabeck, T. Gershon, Ph. Ghez, A. Gianelle, S. Gianì, V. Gibson, L. Giubega, V. V. Gligorov, C. Göbel, D. Golubkov, A. Golutvin, A. Gomes, C. Gotti, M. Grabalosa Gándara, R. Graciani Diaz, L. A. Granado Cardoso, E. Graugés, G. Graziani, A. Grecu, E. Greening, S. Gregson, P. Griffith, L. Grillo, O. Grünberg, B. Gui, E. Gushchin, Yu. Guz, T. Gys, C. Hadjivasiliou, G. Haefeli, C. Haen, S. C. Haines, S. Hall, B. Hamilton, T. Hampson, X. Han, S. Hansmann-Menzemer, N. Harnew, S. T. Harnew, J. Harrison, J. He, T. Head, V. Heijne, K. Hennessy, P. Henrard, L. Henry, J. A. Hernando Morata, E. van Herwijnen, M. Heß, A. Hicheur, D. Hill, M. Hoballah, C. Hombach, W. Hulsbergen, P. Hunt, N. Hussain, D. Hutchcroft, D. Hynds, M. Idzik, P. Ilten, R. Jacobsson, A. Jaeger, J. Jalocha, E. Jans, P. Jaton, A. Jawahery, F. Jing, M. John, D. Johnson, C. R. Jones, C. Joram, B. Jost, N. Jurik, M. Kaballo, S. Kandybei, W. Kanso, M. Karacson, T. M. Karbach, S. Karodia, M. Kelsey, I. R. Kenyon, T. Ketel, B. Khanji, C. Khurewathanakul, S. Klaver, K. Klimaszewski, O. Kochebina, M. Kolpin, I. Komarov, R. F. Koopman, P. Koppenburg, M. Korolev, A. Kozlinskiy, L. Kravchuk, K. Kreplin, M. Kreps, G. Krocker, P. Krokovny, F. Kruse, W. Kucewicz, M. Kucharczyk, V. Kudryavtsev, K. Kurek, T. Kvaratskheliya, V. N. La Thi, D. Lacarrere, G. Lafferty, A. Lai, D. Lambert, R. W. Lambert, G. Lanfranchi, C. Langenbruch, B. Langhans, T. Latham, C. Lazzeroni, R. Le Gac, J. van Leerdam, J.-P. Lees, R. Lefèvre, A. Leflat, J. Lefrançois, S. Leo, O. Leroy, T. Lesiak, B. Leverington, Y. Li, T. Likhomanenko, M. Liles, R. Lindner, C. Linn, F. Lionetto, B. Liu, S. Lohn, I. Longstaff, J. H. Lopes, N. Lopez-March, P. Lowdon, D. Lucchesi, H. Luo, A. Lupato, E. Luppi, O. Lupton, F. Machefert, I. V. Machikhiliyan, F. Maciuc, O. Maev, S. Malde, A. Malinin, G. Manca, G. Mancinelli, A. Mapelli, J. Maratas, J.F. Marchand, U. Marconi, C. Marin Benito, P. Marino, R. Märki, J. Marks, G. Martellotti, A. Martens, A. Martín Sánchez, M. Martinelli, D. Martinez Santos, F. Martinez Vidal, D. Martins Tostes, A. Massafferri, R. Matev, Z. Mathe, C. Matteuzzi, A. Mazurov, M. McCann, J. McCarthy, A. McNab, R. McNulty, B. McSkelly, B. Meadows, F. Meier, M. Meissner, M. Merk, D. A. Milanes, M.-N. Minard, N. Moggi, J. Molina Rodriguez, S. Monteil, M. Morandin, P. Morawski, A. Mordà, M. J. Morello, J. Moron, A.-B. Morris, R. Mountain, F. Muheim, K. Müller, M. Mussini, B. Muster, P. Naik, T. Nakada, R. Nandakumar, I. Nasteva, M. Needham, N. Neri, S. Neubert, N. Neufeld, M. Neuner, A. D. Nguyen, T. D. Nguyen, C. Nguyen-Mau, M. Nicol, V. Niess, R. Niet, N. Nikitin, T. Nikodem, A. Novoselov, D. P. O’Hanlon, A. Oblakowska-Mucha, V. Obraztsov, S. Oggero, S. Ogilvy, O. Okhrimenko, R. Oldeman, G. Onderwater, M. Orlandea, J. M. Otalora Goicochea, P. Owen, A. Oyanguren, B. K. Pal, A. Palano, F. Palombo, M. Palutan, J. Panman, A. Papanestis, M. Pappagallo, L. L. Pappalardo, C. Parkes, C. J. Parkinson, G. Passaleva, G. D. Patel, M. Patel, C. Patrignani, A. Pazos Alvarez, A. Pearce, A. Pellegrino, M. Pepe Altarelli, S. Perazzini, E. Perez Trigo, P. Perret, M. Perrin-Terrin, L. Pescatore, E. Pesen, K. Petridis, A. Petrolini, E. Picatoste Olloqui, B. Pietrzyk, T. Pilař, D. Pinci, A. Pistone, S. Playfer, M. Plo Casasus, F. Polci, A. Poluektov, E. Polycarpo, A. Popov, D. Popov, B. Popovici, C. Potterat, E. Price, J.D. Price, J. Prisciandaro, A. Pritchard, C. Prouve, V. Pugatch, A. Puig Navarro, G. Punzi, W. Qian, B. Rachwal, J. H. Rademacker, B. Rakotomiaramanana, M. Rama, M. S. Rangel, I. Raniuk, N. Rauschmayr, G. Raven, F. Redi, S. Reichert, M. M. Reid, A. C. dos Reis, S. Ricciardi, S. Richards, M. Rihl, K. Rinnert, V. Rives Molina, P. Robbe, A. B. Rodrigues, E. Rodrigues, P. Rodriguez Perez, S. Roiser, V. Romanovsky, A. Romero Vidal, M. Rotondo, J. Rouvinet, T. Ruf, H. Ruiz, P. Ruiz Valls, J. J. Saborido Silva, N. Sagidova, P. Sail, B. Saitta, V. Salustino Guimaraes, C. Sanchez Mayordomo, B. Sanmartin Sedes, R. Santacesaria, C. Santamarina Rios, E. Santovetti, A. Sarti, C. Satriano, A. Satta, D.M. Saunders, M. Savrie, D. Savrina, M. Schiller, H. Schindler, M. Schlupp, M. Schmelling, B. Schmidt, O. Schneider, A. Schopper, M.-H. Schune, R. Schwemmer, B. Sciascia, A. Sciubba, M. Seco, A. Semennikov, I. Sepp, N. Serra, J. Serrano, L. Sestini, P. Seyfert, M. Shapkin, I. Shapoval, Y. Shcheglov, T. Shears, L. Shekhtman, V. Shevchenko, A. Shires, R. Silva Coutinho, G. Simi, M. Sirendi, N. Skidmore, T. Skwarnicki, N. A. Smith, E. Smith, E. Smith, J. Smith, M. Smith, H. Snoek, M. D. Sokoloff, F. J. P. Soler, F. Soomro, D. Souza, B. Souza De Paula, B. Spaan, A. Sparkes, P. Spradlin, S. Sridharan, F. Stagni, M. Stahl, S. Stahl, O. Steinkamp, O. Stenyakin, S. Stevenson, S. Stoica, S. Stone, B. Storaci, S. Stracka, M. Straticiuc, U. Straumann, R. Stroili, V. K. Subbiah, L. Sun, W. Sutcliffe, K. Swientek, S. Swientek, V. Syropoulos, M. Szczekowski, P. Szczypka, D. Szilard, T. Szumlak, S. T’Jampens, M. Teklishyn, G. Tellarini, F. Teubert, C. Thomas, E. Thomas, J. van Tilburg, V. Tisserand, M. Tobin, S. Tolk, L. Tomassetti, D. Tonelli, S. Topp-Joergensen, N. Torr, E. Tournefier, S. Tourneur, M. T. Tran, M. Tresch, A. Tsaregorodtsev, P. Tsopelas, N. Tuning, M. Ubeda Garcia, A. Ukleja, A. Ustyuzhanin, U. Uwer, C. Vacca, V. Vagnoni, G. Valenti, A. Vallier, R. Vazquez Gomez, P. Vazquez Regueiro, C. Vázquez Sierra, S. Vecchi, J. J. Velthuis, M. Veltri, G. Veneziano, M. Vesterinen, B. Viaud, D. Vieira, M. Vieites Diaz, X. Vilasis-Cardona, A. Vollhardt, D. Volyanskyy, D. Voong, A. Vorobyev, V. Vorobyev, C. Voß, H. Voss, J. A. de Vries, R. Waldi, C. Wallace, R. Wallace, J. Walsh, S. Wandernoth, J. Wang, D. R. Ward, N. K. Watson, D. Websdale, M. Whitehead, J. Wicht, D. Wiedner, G. Wilkinson, M. P. Williams, M. Williams, H.W. Wilschut, F. F. Wilson, J. Wimberley, J. Wishahi, W. Wislicki, M. Witek, G. Wormser, S. A. Wotton, S. Wright, K. Wyllie, Y. Xie, Z. Xing, Z. Xu, Z. Yang, X. Yuan, O. Yushchenko, M. Zangoli, M. Zavertyaev, L. Zhang, W. C. Zhang, Y. Zhang, A. Zhelezov, A. Zhokhov, L. Zhong, A. Zvyagin

**Affiliations:** Centro Brasileiro de Pesquisas Físicas (CBPF), Rio de Janeiro, Brazil; Universidade Federal do Rio de Janeiro (UFRJ), Rio de Janeiro, Brazil; Center for High Energy Physics, Tsinghua University, Beijing, China; LAPP, Université de Savoie, CNRS/IN2P3, Annecy-Le-Vieux, France; LPC, Clermont Université, Université Blaise Pascal, CNRS/IN2P3, Clermont-Ferrand, France; CPPM, Aix-Marseille Université, CNRS/IN2P3, Marseille, France; LAL, Université Paris-Sud, CNRS/IN2P3, Orsay, France; LPNHE, Université Pierre et Marie Curie, Université Paris Diderot, CNRS/IN2P3, Paris, France; Fakultät Physik, Technische Universität Dortmund, Dortmund, Germany; Max-Planck-Institut für Kernphysik (MPIK), Heidelberg, Germany; Physikalisches Institut, Ruprecht-Karls-Universität Heidelberg, Heidelberg, Germany; School of Physics, University College Dublin, Dublin, Ireland; Sezione INFN di Bari, Bari, Italy; Sezione INFN di Bologna, Bologna, Italy; Sezione INFN di Cagliari, Cagliari, Italy; Sezione INFN di Ferrara, Ferrara, Italy; Sezione INFN di Firenze, Florence, Italy; Laboratori Nazionali dell’INFN di Frascati, Frascati, Italy; Sezione INFN di Genova, Genoa, Italy; Sezione INFN di Milano Bicocca, Milan, Italy; Sezione INFN di Milano, Milan, Italy; Sezione INFN di Padova, Padua, Italy; Sezione INFN di Pisa, Pisa, Italy; Sezione INFN di Roma Tor Vergata, Rome, Italy; Sezione INFN di Roma La Sapienza, Rome, Italy; Henryk Niewodniczanski Institute of Nuclear Physics, Polish Academy of Sciences, Kraków, Poland; Faculty of Physics and Applied Computer Science, AGH-University of Science and Technology, Kraków, Poland; National Center for Nuclear Research (NCBJ), Warsaw, Poland; Horia Hulubei National Institute of Physics and Nuclear Engineering, Bucharest-Magurele, Romania; Petersburg Nuclear Physics Institute (PNPI), Gatchina, Russia; Institute of Theoretical and Experimental Physics (ITEP), Moscow, Russia; Institute of Nuclear Physics, Moscow State University (SINP MSU), Moscow, Russia; Institute for Nuclear Research of the Russian Academy of Sciences (INR RAN), Moscow, Russia; Budker Institute of Nuclear Physics (SB RAS) and Novosibirsk State University, Novosibirsk, Russia; Institute for High Energy Physics (IHEP), Protvino, Russia; Universitat de Barcelona, Barcelona, Spain; Universidad de Santiago de Compostela, Santiago de Compostela, Spain; European Organization for Nuclear Research (CERN), Geneva, Switzerland; Ecole Polytechnique Fédérale de Lausanne (EPFL), Lausanne, Switzerland; Physik-Institut, Universität Zürich, Zurich, Switzerland; Nikhef National Institute for Subatomic Physics, Amsterdam, The Netherlands; Nikhef National Institute for Subatomic Physics, VU University Amsterdam, Amsterdam, The Netherlands; NSC Kharkiv Institute of Physics and Technology (NSC KIPT), Kharkiv, Ukraine; Institute for Nuclear Research of the National Academy of Sciences (KINR), Kyiv, Ukraine; University of Birmingham, Birmingham, UK; H.H. Wills Physics Laboratory, University of Bristol, Bristol, UK; Cavendish Laboratory, University of Cambridge, Cambridge, UK; Department of Physics, University of Warwick, Coventry, UK; STFC Rutherford Appleton Laboratory, Didcot, UK; School of Physics and Astronomy, University of Edinburgh, Edinburgh, UK; School of Physics and Astronomy, University of Glasgow, Glasgow, UK; Oliver Lodge Laboratory, University of Liverpool, Liverpool, UK; Imperial College London, London, UK; School of Physics and Astronomy, University of Manchester, Manchester, UK; Department of Physics, University of Oxford, Oxford, UK; Massachusetts Institute of Technology, Cambridge, MA USA; University of Cincinnati, Cincinnati, OH USA; University of Maryland, College Park, MD USA; Syracuse University, Syracuse, NY USA; Pontifícia Universidade Católica do Rio de Janeiro (PUC-Rio), Rio de Janeiro, Brazil; Institute of Particle Physics, Central China Normal University, Wuhan, Hubei China; Institut für Physik, Universität Rostock, Rostock, Germany; National Research Centre Kurchatov Institute, Moscow, Russia; Instituto de Fisica Corpuscular (IFIC), Universitat de Valencia-CSIC, Valencia, Spain; KVI, University of Groningen, Groningen, The Netherlands; Celal Bayar University, Manisa, Turkey

## Abstract

The production of the $$\eta _c (1S)$$ state in proton-proton collisions is probed via its decay to the $$p\overline{p}$$ final state with the LHCb detector, in the rapidity range $$2.0 < y < 4.5$$ and in the meson transverse-momentum range $$p_\mathrm{T} > 6.5 \mathrm{{\,GeV/}{ c}} $$. The cross-section for prompt production of $$\eta _c (1S)$$ mesons relative to the prompt $${{ J}}/{\psi } $$ cross-section is measured, for the first time, to be $$\sigma _{\eta _c (1S)}/\sigma _{{{{ J}}/{\psi }}} = 1.74\, \pm \,0.29\, \pm \, 0.28\, \pm \,0.18 _{{\mathcal{B}}}$$ at a centre-of-mass energy $${\sqrt{s}} = 7 {~\mathrm{TeV}}$$ using data corresponding to an integrated luminosity of 0.7 fb$$^{-1}$$, and $$\sigma _{\eta _c (1S)}/\sigma _{{{{ J}}/{\psi }}} = 1.60 \pm 0.29 \pm 0.25 \pm 0.17 _{{\mathcal{B}}}$$ at $${\sqrt{s}} = 8 {~\mathrm{TeV}}$$ using 2.0 fb$$^{-1}$$. The uncertainties quoted are, in order, statistical, systematic, and that on the ratio of branching fractions of the $$\eta _c (1S)$$ and $${{ J}}/{\psi } $$ decays to the $$p\overline{p}$$ final state. In addition, the inclusive branching fraction of $${b} $$-hadron decays into $$\eta _c (1S)$$ mesons is measured, for the first time, to be $${\mathcal{B}}( b {\rightarrow } \eta _c X ) = (4.88\, \pm \,0.64\, \pm \,0.29\, \pm \, 0.67 _{{\mathcal{B}}}) \times 10^{-3}$$, where the third uncertainty includes also the uncertainty on the $${{ J}}/{\psi } $$ inclusive branching fraction from $${b} $$-hadron decays. The difference between the $${{ J}}/{\psi } $$ and $$\eta _c (1S)$$ meson masses is determined to be $$114.7 \pm 1.5 \pm 0.1 {\mathrm {\,MeV\!/}c^2} $$.

## Introduction

High centre-of-mass energies available in proton-proton collisions at the LHC allow models describing charmonium production to be tested. We distinguish promptly produced charmonia from those originating from $${b} $$-hadron decays. Promptly produced charmonia include charmonia directly produced in parton interactions and those originating from the decays of heavier quarkonium states, which are in turn produced in parton interactions. While measurements of $${{ J}}/{\psi } $$ and $${\psi } {(2S)}$$ meson production rates at the LHC  [1–6] are successfully described by next-to-leading order (NLO) calculations in non-relativistic quantum chromodynamics (QCD) [7], the observation of small or no polarization in $${{ J}}/{\psi } $$ meson prompt production [2] remains unexplained within the available theoretical framework [8]. The investigation of the lowest state, the $$\eta _c (1S)$$ meson, can provide important additional information on the long-distance matrix elements [9, 10]. In particular, the heavy-quark spin-symmetry relation between the $$\eta _c (1S)$$ and $${{ J}}/{\psi } $$ matrix elements can be tested, with the NLO calculations predicting a different dependence of the production rates on charmonium transverse momentum, $$p_\mathrm{T}$$, for spin singlet ($$\eta _c (1S)$$) and triplet ($${{ J}}/{\psi } $$, $$\chi _{cJ}$$) states [11–13]. Thus, a measurement of the $$p_\mathrm{T}$$ dependence of the $$\eta _c (1S)$$ production rate, in particular in the low $$p_\mathrm{T}$$ region, can have important implications. Recent LHCb results on prompt production of $$\upchi _{c}$$ states [14] provide information on the production of the *P*-wave states $$\upchi _{{{c}} 0}$$ and $$\upchi _{{{c}} 2}$$ at low $$p_\mathrm{T}$$, using the well-understood $$\upchi _{{{c}} 1}$$ production as a reference. A measurement of the cross-section of prompt $$\eta _c (1S)$$ production may allow an important comparison with the $$\upchi _{{{c}} 0}$$ results and yields indirect information on the production of heavier states.

At LHC energies, all $${b} $$-hadron species are produced, including weakly decaying $${{B}} ^-$$, $${\overline{{B}}{}} {}^0$$, $${\overline{{B}}{}} {}^0_{\mathrm {s}} $$, $${{B}} _{{c}} ^-$$ mesons, $${b} $$-baryons, and their charge-conjugate states. A previous study of inclusive $$\eta _c (1S)$$ meson production in $${b} $$-hadron decays by the CLEO experiment, based on a sample of $${{B}} ^-$$ and $${\overline{{B}}{}} {}^0$$ mesons, placed an upper limit on the combined inclusive branching fraction of $${{B}} ^-$$ and $${\overline{{B}}{}} {}^0$$ meson decays into final states containing an $$\eta _c (1S)$$ meson of $${\mathcal{B}}( {{{B}} ^-}, {{\overline{{B}}{}} {}^0} {\rightarrow } \eta _c (1S) X ) < 9 \times 10^{-3}$$ at $$90 \%$$ confidence level [15]. Exclusive analyses of $$\eta _c (1S)$$ and $${{ J}}/{\psi } $$ meson production in $${b} $$-hadron decays using the $${{B}} {\rightarrow } K ( {{{ p}}} \overline{{{{ p}}}})$$ decay mode have been performed by the BaBar experiment [16], by the Belle experiment [17] and recently by the LHCb experiment [18].

In the present paper we report the first measurement of the cross-section for the prompt production of $$\eta _c (1S)$$ mesons in $${{ p}} $$$${{ p}} $$ collisions at $${\sqrt{s}} = 7 {~\mathrm{TeV}}$$ and $${\sqrt{s}} = 8 {~\mathrm{TeV}}$$ centre-of-mass energies, as well as the $${b} $$-hadron inclusive branching fraction into $$\eta _c (1S)$$ final states. This paper extends the scope of previous charmonium production studies reported by LHCb, which were restricted to the use of $${{ J}}/{\psi } $$ or $${\psi } {(2S)}$$ decays to dimuon final states [1, 2, 14, 19]. In order to explore states that do not have $$J^{PC} = 1^{--}$$ quantum numbers, while avoiding reconstruction of radiative decays with low-energy photons, the authors of Ref. [20] suggested to investigate hadronic final states. In the present analysis, we reconstruct $$\eta _c (1S)$$ mesons decaying into the $$p\overline{p}$$ final state. All well-established charmonium states decay to $$p\overline{p}$$ final states [20, 21]. With its powerful charged-hadron identification and high charmonium production rate, the LHCb experiment is well positioned for these studies. The measurements are performed relative to the topologically and kinematically similar $${{{ J}}/{\psi }} {\rightarrow } {{{ p}}} \overline{{{{ p}}}} $$ channel, which allows partial cancellation of systematic uncertainties in the ratio. This is the first such inclusive analysis using decays to hadronic final states performed at a hadron collider.

In addition, a departure in excess of two standard deviations between the recent BES III results [22, 23] and earlier measurements [21] motivates the determination of the difference between $${{ J}}/{\psi } $$ and $$\eta _c (1S)$$ meson masses $$\Delta M _{{{{ J}}/{\psi }}, \, \eta _c (1S)} \equiv M_{{{{ J}}/{\psi }}} - M_{\eta _c (1S)}$$ using a different technique and final state. In the present analysis, the low-background sample of charmonia produced in $${b} $$-hadron decays is used to determine $$\Delta M _{{{{ J}}/{\psi }}, \, \eta _c (1S)}$$ and the $$\eta _c (1S)$$ natural width, $$\Gamma _{\eta _c (1S)}$$.

In Sect. 2 we present the LHCb detector and data sample used for the analysis. Section 3 describes the analysis details, while the systematic uncertainties are discussed in Sect. 4. The results are given in Sect. 5 and summarized in Sect. 6.

## LHCb detector and data sample

The LHCb detector [24] is a single-arm forward spectrometer covering the pseudorapidity range $$2< \eta <5$$, designed for the study of particles containing $${b} $$ or $${c} $$ quarks. The detector includes a high-precision tracking system consisting of a silicon-strip vertex detector surrounding the *pp* interaction region, a large-area silicon-strip detector located upstream of a dipole magnet with a bending power of about 4 $$\mathrm{\,Tm}$$, and three stations of silicon-strip detectors and straw drift tubes placed downstream of the magnet. The combined tracking system provides a momentum measurement with a relative uncertainty that varies from 0.4 % at low momentum to 0.6% at 100 $$\mathrm{{\,GeV/}{ c}}$$, and an impact parameter measurement with a resolution of 20$${\,{\upmu}{\mathrm m}}$$ for charged particles with large transverse momentum. Different types of charged hadrons are distinguished using information from two ring-imaging Cherenkov detectors. Photon, electron, and hadron candidates are identified by a system consisting of scintillating-pad and preshower detectors, an electromagnetic calorimeter, and a hadronic calorimeter. Muons are identified by a system composed of alternating layers of iron and multiwire proportional chambers. The trigger consists of a hardware stage, based on information from the calorimeter and muon systems, followed by a software stage, which applies a full event reconstruction.

Events enriched in signal decays are selected by the hardware trigger, based on the presence of a single high-energy deposit in the calorimeter. The subsequent software trigger specifically rejects high-multiplicity events and selects events with two oppositely charged particles having good track-fit quality and transverse momentum larger than 1.9$$\mathrm{{\,GeV/}{ c}}$$. Proton and antiproton candidates are identified using the information from Cherenkov and tracking detectors [25]. Selected $${{ p}} $$ and $$\overline{{{{ p}}}}$$ candidates are required to form a good quality vertex. In order to further suppress the dominant background from accidental combinations of random tracks (combinatorial background), charmonium candidates are required to have high transverse momentum, $$p_\mathrm{T} > 6.5 \mathrm{{\,GeV/}{ c}} $$.

The present analysis uses $${{ p}} $$$${{ p}} $$ collision data recorded by the LHCb experiment at $${\sqrt{s}} = 7 {~\mathrm{TeV}}$$, corresponding to an integrated luminosity of 0.7 fb$$^{-1}$$, and at $${\sqrt{s}} = 8 {~\mathrm{TeV}}$$, corresponding to an integrated luminosity of 2.0 fb$$^{-1}$$.

Simulated samples of $$\eta _c (1S)$$ and $${{ J}}/{\psi } $$ mesons decaying to the $$p\overline{p}$$ final state, and $${{ J}}/{\psi } $$ decaying to the $${{p}} $$$$\overline{{{{p}}}}$$$${\pi } ^0$$ final state, are used to estimate efficiency ratios, the contribution from the decay $${{{ J}}/{\psi }} {\rightarrow } {{{ p}}} \overline{{{{ p}}}} {{\pi } ^0} $$, and to evaluate systematic uncertainties. In the simulation, *pp* collisions are generated using Pythia  [26] with a specific LHCb configuration [27]. Decays of hadronic particles are described by EvtGen  [28], in which final-state radiation is generated using Photos  [29]. The interaction of the generated particles with the detector and its response are implemented using the Geant4 toolkit [30, 31] as described in Ref. [32].

## Signal selection and data analysis

The signal selection is largely performed at the trigger level. The offline analysis, in addition, requires the transverse momentum of $${{ p}} $$ and $$\overline{{{{ p}}}}$$ to be $$p_\mathrm{T} > 2.0 \mathrm{{\,GeV/}{ c}} $$, and restricts charmonium candidates to the rapidity range $$2.0 < y < 4.5$$.

Discrimination between promptly produced charmonium candidates and those from *b*-hadron decays is achieved using the pseudo-decay time $$t_{z} = \Delta z \cdot M / p_z$$, where $$\Delta z$$ is the distance along the beam axis between the corresponding $${{ p}} $$$${{ p}} $$ collision vertex (primary vertex) and the candidate decay vertex, *M* is the candidate mass, and $$p_z$$ is the longitudinal component of its momentum. Candidates with $$t_{z} < 80 \mathrm \,fs $$ are classified as prompt, while those with $$t_{z} > 80 \mathrm \,fs $$ are classified as having originated from $${b} $$-hadron decays. For charmonium candidates from $${b} $$-hadron decays, a significant displacement of the proton tracks with respect to the primary vertex is also required.

The selected samples of prompt charmonium candidates and charmonia from $${b} $$-hadron decays have some candidates wrongly classified (cross-feed). The cross-feed probability is estimated using simulated samples and is scaled using the observed signal candidate yields in data. The cross-feed component is subtracted to obtain the ratio of produced $$\eta _c (1S)$$ and $${{ J}}/{\psi } $$ mesons decaying into the $$p\overline{p}$$ final state. Corrections range from 2% to 3% for the ratio of promptly produced $$\eta _c (1S)$$ and $${{ J}}/{\psi } $$ mesons, and from 8% to 10% for the ratio of charmonia produced in $${b} $$-hadron decays.

The ratios of signal yields are expressed in terms of ratios of cross-sections multiplied by the decay branching fractions as

$$\begin{aligned} \frac{N^{P}_{\eta _c (1S)}}{N^{P}_{{{{ J}}/{\psi }}}}&= \frac{\sigma (\eta _c (1S)) \times {\mathcal{B}}( \eta _c (1S) {\rightarrow } {{{ p}}} \overline{{{{ p}}}})}{\sigma ({{{ J}}/{\psi }}) \times {\mathcal{B}}( {{{ J}}/{\psi }} {\rightarrow } {{{ p}}} \overline{{{{ p}}}})}, \\ \frac{N^{b}_{\eta _c (1S)}}{N^{b}_{{{{ J}}/{\psi }}}}&= \frac{{\mathcal{B}}( {{b}} {\rightarrow } \eta _c (1S) X ) \times {\mathcal{B}}( \eta _c (1S) {\rightarrow } {{{ p}}} \overline{{{{ p}}}})}{{\mathcal{B}}( {{b}} {\rightarrow } {{{ J}}/{\psi }} X ) \times {\mathcal{B}}( {{{ J}}/{\psi }} {\rightarrow } {{{ p}}} \overline{{{{ p}}}})}, \end{aligned}$$

where $$N^P$$ and $$N^b$$ are the numbers of charmonia from prompt production and $${b} $$-hadron decays, respectively. The simulation describes the kinematic-related differences between the $$\eta _c (1S)$$ and $${{ J}}/{\psi } $$ decay modes reasonably well and predicts that the relative efficiencies for selecting and reconstructing $$\eta _c (1S)$$ and $${{ J}}/{\psi } $$ mesons differ by less than 0.5%. Equal efficiencies are assumed for the $$\eta _c (1S)$$ and $${{ J}}/{\psi } $$ meson reconstruction and selection criteria. The efficiency for selecting and reconstructing prompt $${{ J}}/{\psi } $$ mesons is corrected for polarization effects, as a function of rapidity and $$p_\mathrm{T}$$, according to Ref. [2].

The numbers of reconstructed $$\eta _c (1S)$$ and $${{ J}}/{\psi } $$ candidates are extracted from an extended maximum likelihood fit to the unbinned $$p\overline{p}$$ invariant mass distribution. The $${{ J}}/{\psi } $$ peak position $$M_{{{{ J}}/{\psi }}}$$ and the mass difference $$\Delta M_{{{{ J}}/{\psi }}, \eta _c (1S)}$$ are fitted in the sample of charmonia from $${b} $$-hadron decays, where the signal is more prominent because of the reduced background level due to charmonium decay-vertex displacement requirements. The results are then used to apply Gaussian constraints in the fit to the $$p\overline{p}$$ invariant mass spectrum in the prompt production analysis, where the signal-to-background ratio is smaller, due to large combinatorial backgrounds.

The signal shape is defined by the detector response, combined with the natural width in the case of the $$\eta _c (1S)$$ resonance. The detector response is described using two Gaussian functions with a common mean value. In the description of each resonance, the ratio of narrow to wide Gaussian widths, $$\sigma ^a_{{{{ J}}/{\psi }}} / \sigma ^b_{{{{ J}}/{\psi }}} = \sigma ^a_{\eta _c (1S)} / \sigma ^b_{\eta _c (1S)}$$, the fraction of the narrow Gaussian component, and the ratio of the $$\eta _c (1S)$$ and $${{ J}}/{\psi } $$ narrow Gaussian widths, $$\sigma ^a_{\eta _c (1S)}/\sigma ^a_{{{{ J}}/{\psi }}}$$, are fixed in the fit to the values observed in simulation. The only resolution parameter left free in the fit to the low-background sample from $${b} $$-hadron decays, $$\sigma ^a_{{{{ J}}/{\psi }}}$$, is fixed to its central value in the fit to the prompt sample. The natural width $$\Gamma _{\eta _c (1S)}$$ of the $$\eta _c (1S)$$ resonance is also extracted from the fit to the $${b} $$-hadron decays sample, and is fixed to that value in the prompt production analysis. Gaussian constraints on the $${{ J}}/{\psi } $$ meson mass and the $$\Delta M_{{{{ J}}/{\psi }}, \, \eta _c (1S)}$$ mass difference from the fit to the $${b} $$-hadron decays sample are applied in the prompt production analysis. The fit with free mass values gives consistent results.

The combinatorial background is parametrized by an exponential function in the fit of the sample from $${b} $$-hadron decays, and by a third-order polynomial in the fit to the prompt sample.

Combinations of $p\overline{p}$ from the decay ${{{ J}}/{\psi }} {\rightarrow } {{{ p}}} \overline{{{{ p}}}} {{\pi } ^0}$ potentially affect the region close to the $\eta _c (1S)$ signal; hence, this contribution is specifically included in the background description. It produces a non-peaking contribution, and its mass distribution is described by a square-root shape to account for the phase space available to the $p\overline{p}$ system from the ${{{ J}}/{\psi }} {\rightarrow } {{{ p}}} \overline{{{{ p}}}} {{\pi } ^0}$ decay, convolved with two Gaussian functions to account for the detector mass resolution. In the fit to the $p\overline{p}$ invariant mass spectrum, the normalization of this contribution is fixed using the number of candidates found in the ${{ J}}/{\psi }$ signal peak and the ratios of branching fractions and efficiencies for the ${{{ J}}/{\psi }} {\rightarrow } {{{ p}}} \overline{{{{ p}}}} {{\pi } ^0}$ and ${{{ J}}/{\psi }} {\rightarrow } {{{ p}}} \overline{{{{ p}}}}$ decay modes.

**Fig. 1:**
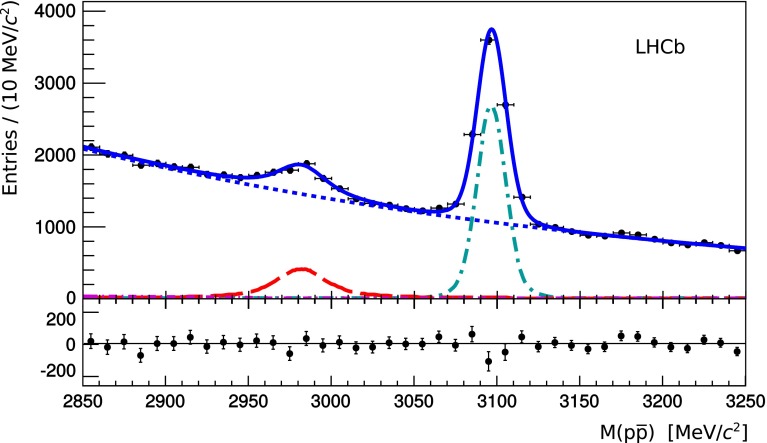
Proton–antiproton invariant mass spectrum for candidates originating from a secondary vertex and reconstructed in ${\sqrt{s}} = 7 {~\mathrm{TeV}}$ and ${\sqrt{s}} = 8 {~\mathrm{TeV}}$ data. The *solid blue line* represents the best-fit curve, the *long-dashed red line* corresponds to the $\eta _c (1S)$ signal, the *dashed-dotted cyan line* corresponds to the ${{ J}}/{\psi }$ signal, and the *dashed magenta line* corresponds to the small contribution from ${{{ J}}/{\psi }} {\rightarrow } {{{ p}}} \overline{{{{ p}}}} {{\pi } ^0}$ decays with the pion unreconstructed. The *dotted blue line* corresponds to the combinatorial background. The distribution of the difference between data points and the fit function is shown in the *bottom panel*

The $$p\overline{p}$$ invariant mass spectra for charmonium candidates from $${b} $$-hadron decays in the 7 $$~\mathrm{TeV}$$and 8 $$~\mathrm{TeV}$$data are observed to be consistent. The two data samples are therefore combined and the resulting spectrum is shown in Fig. 1 with the fit overlaid.

The $${{ J}}/{\psi } $$ meson signal is modelled using a double-Gaussian function. The $$\eta _c (1S)$$ signal is modelled using a relativistic Breit–Wigner function convolved with a double-Gaussian function. The background contribution from the $${{{ J}}/{\psi }} {\rightarrow } {{{ p}}} \overline{{{{ p}}}} {{\pi } ^0} $$ decay with an unreconstructed pion, is small. The fit yields $$2020 \pm 230$$$$\eta _c (1S)$$ signal decays and $$6110 \pm 116$$$${{ J}}/{\psi } $$ signal decays.

**Fig. 2:**
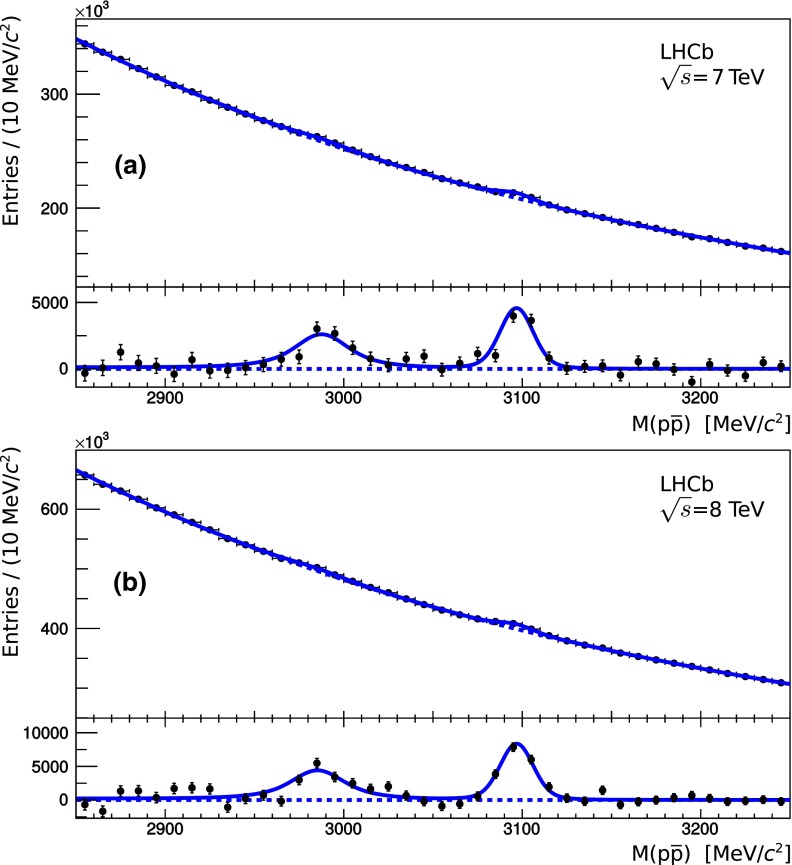
Proton–antiproton invariant mass spectrum for candidates originating from a primary vertex (*upper panel* in each plot), and distribution of differences between data and the background distribution resulting from the fit (*lower panel* in each plot), in data at **a** ${\sqrt{s}} = 7 {~\mathrm{TeV}}$ and **b** ${\sqrt{s}} = 8 {~\mathrm{TeV}}$ centre-of-mass energies. Distributions on the *upper panels* are zero-suppressed

The results of the fit to the $$p\overline{p}$$ invariant mass spectrum of the prompt sample are shown in Fig. 2a and b for data collected at $${\sqrt{s}} = 7{~\mathrm{TeV}}$$ and $${\sqrt{s}} = 8{~\mathrm{TeV}}$$, respectively. The fits yield $$13 \, 370 \pm 2260$$$$\eta _c (1S)$$ and $$11 \, 052 \pm 1004$$$${{ J}}/{\psi } $$ signal decays for the data taken at $${\sqrt{s}} = 7{~\mathrm{TeV}}$$, and $$22 \, 416 \pm 4072$$$$\eta _c (1S)$$ and $$20 \, 217 \pm 1403$$$${{ J}}/{\psi } $$ signal decays for the $${\sqrt{s}} = 8{~\mathrm{TeV}}$$ data.

In order to assess the quality of these unbinned fits to the invariant $p\overline{p}$ mass spectra, the chisquare per degree of freedom was calculated for the binning schemes shown in Figs. 1, and 2a, b. The values are 1.3, 1.7 and 1.8, respectively.

From the observed $\eta _c (1S)$ and ${{ J}}/{\psi }$ yields, and taking into account cross-feed between the samples, the yield ratios are obtained as

$$\begin{aligned} ( N^{P}_{\eta _c (1S)}/N^{P}_{{{{ J}}/{\psi }}} )_{{\sqrt{s}} = 7{~\mathrm{TeV}}}&= 1.24 \pm 0.21 , \\ ( N^{P}_{\eta _c (1S)}/N^{P}_{{{{ J}}/{\psi }}} )_{{\sqrt{s}} = 8{~\mathrm{TeV}}}&= 1.14 \pm 0.21 \end{aligned}$$

and

$$\begin{aligned} N^{b}_{\eta _c (1S)}/N^{b}_{{{{ J}}/{\psi }}} = 0.302 \pm 0.039 \end{aligned}$$

for the prompt production and charmonium production in $${b} $$-hadron decays. Only statistical uncertainties are given in the above ratios.

## Systematic uncertainties

We consider systematic uncertainties due to limited knowledge of the detector mass resolution, the $${{ J}}/{\psi } $$ polarization, the $$\eta _c (1S)$$ natural width, possible differences of the prompt charmonium production spectra in data and simulation, cross-feed between the prompt charmonium sample and the charmonium sample from $${b} $$-hadron decays, background description and feed-down from $${{{ J}}/{\psi }} {\rightarrow } {{{ p}}} \overline{{{{ p}}}} {{\pi } ^0} $$ decays.

**Table 1:** Summary of uncertainties for the yield ratio $N_{\eta _c (1S)} / N_{{{{ J}}/{\psi }}}$

	Production in	Prompt production	
	$${b} $$-Hadron decays	$${\sqrt{s}} = 7{~\mathrm{TeV}}$$	$${\sqrt{s}} = 8{~\mathrm{TeV}}$$
Statistical uncertainty	0.039	0.21	0.21
Systematic uncertainties			
Signal resolution ratio (simulation)	0.006	0.04	0.03
Signal resolution variation		0.01	0.01
${{ J}}/{\psi }$ polarization	0.009	0.02	0.02
$\Gamma _{\eta _c (1S)}$ variation		0.15	0.14
Prompt production spectrum	0.003	0.07	0.06
Cross-feed	0.008	0.01	0.01
Background model	0.011	0.09	0.09
Total systematic uncertainty	0.018	0.20	0.18

Uncertainties due to limited knowledge of the detector mass resolution are estimated by assigning the same $$\sigma ^a$$ value to the $$\eta _c (1S)$$ and $${{ J}}/{\psi } $$ signal description for the $${b} $$-hadron sample, and by varying the $$\sigma ^a$$ parameters in the prompt production analysis within their uncertainties. Uncertainties associated with the $${{ J}}/{\psi } $$ polarization in the prompt production reflect those of the polarization measurement in Ref. [2]. We evaluate a potential contribution from $${ J}/{\psi }$$ polarization in *b*-hadron decays using a BaBar study [32] of the $${{ J}}/{\psi } $$ polarization in inclusive decays of *B* mesons. Simulations are used to estimate the effective polarization parameter for the LHCb kinematic region where the *b*-hadrons have a high boost and the longitudinal polarization is significantly reduced. A conservative value for the polarization parameter of −0.2 is used to estimate the corresponding systematic uncertainty. In order to estimate the systematic uncertainty associated with the $$\eta _c (1S)$$ natural width, which enters the results for the prompt production analysis, the world average $$\Gamma _{\eta _c (1S)}$$ value of $$32.0~ \mathrm {\,MeV} $$ from Ref. [21] is used. Possible differences of the prompt charmonium production spectra in data and simulation are estimated by correcting the efficiency derived from simulation according to the observed $$p_\mathrm{T}$$ distribution. The uncertainty related to the cross-feed is estimated by varying the signal yields in each sample according to their uncertainties. Uncertainties associated with the background description are estimated by using an alternative parametrization and varying the fit range. The uncertainty due to the contribution from the $${{{ J}}/{\psi }} {\rightarrow } {{{ p}}} \overline{{{{ p}}}} {{\pi } ^0} $$ decay is dominated by the modelling of the $${{{ p}}} \overline{{{{ p}}}} $$ invariant mass shape, and is estimated by using an alternative parametrization, which is linear instead of the square root. Possible systematic effect related to separation between prompt and $${b} $$-decays samples, was checked by varying the $$t_z$$ discriminant value from 80 to $$120 ~\mathrm \,fs $$. The results are found to be stable under variation of the value of the $$t_z$$ discriminant, and no related systematic uncertainty is assigned. Table 1 lists the systematic uncertainties for the production yield ratio. The total systematic uncertainty is estimated as the quadratic sum of the uncertainties from the sources listed in Table 1 and, in the case of the prompt production measurement, is dominated by the uncertainty associated with the $$\eta _c (1S)$$ natural width. For the measurement with *b*-hadron decays the uncertainties associated with the background model, the $${{ J}}/{\psi }$$ polarization and the cross-feed provide significant contributions.

## Results

The yield ratio for charmonium production in $${b} $$-hadron decays is obtained as

$$\begin{aligned} N^{b}_{\eta _c (1S)}/N^{b}_{{{{ J}}/{\psi }}} = 0.302 \pm 0.039 \pm 0.015. \end{aligned}$$

In all quoted results, the first uncertainty refers to the statistical contribution and the second to the systematic contribution. By correcting the yield ratio with the ratio of branching fractions $${\mathcal{B}}( {{{ J}}/{\psi }} {\rightarrow } {{{ p}}} \overline{{{{ p}}}}) / {\mathcal{B}}( \eta _c (1S) {\rightarrow } {{{ p}}} \overline{{{{ p}}}}) = 1.39 \pm 0.15$$ [21], the ratio of the inclusive $${b} $$-hadron branching fractions into $$\eta _c (1S)$$ and $${{ J}}/{\psi } $$ final states for charmonium transverse momentum $$p_\mathrm{T} > 6.5 \mathrm{{\,GeV/}{ c}} $$ is measured to be

$$\begin{aligned} {\mathcal{B}}( {{b}} {\rightarrow } \eta _c (1S) X ) / {\mathcal{B}}( {{b}} {\rightarrow } {{{ J}}/{\psi }} X ) = 0.421 \pm 0.055 \pm 0.025 \pm 0.045_{{\mathcal{B}}} , \end{aligned}$$

where the third uncertainty is due to that on the $${{{ J}}/{\psi }} {\rightarrow } {{{ p}}} \overline{{{{ p}}}} $$ and $$\eta _c (1S) {\rightarrow } {{{ p}}} \overline{{{{ p}}}} $$ branching fractions [21]. Assuming that the $$p_\mathrm{T} > 6.5 \mathrm{{\,GeV/}{ c}} $$ requirement does not bias the distribution of charmonium momentum in the $${b} $$-hadron rest frame, and using the branching fraction of $${b} $$-hadron inclusive decays into $${{ J}}/{\psi } $$ mesons from Ref. [21], $${\mathcal{B}}( {{b}} {\rightarrow } {{{ J}}/{\psi }} X ) = ( 1.16 \pm 0.10)\%$$, the inclusive branching fraction of $$\eta _c (1S)$$ from $${b} $$-hadron decays is derived as

$$\begin{aligned} {\mathcal{B}}( b {\rightarrow } \eta _c (1S) X ) = (4.88 \pm 0.64 \pm 0.29 \pm 0.67 _{{\mathcal{B}}}) \times 10^{-3}, \end{aligned}$$

where the third uncertainty component includes also the uncertainty on the $${{ J}}/{\psi } $$ inclusive branching fraction from $${b} $$-hadron decays. This is the first measurement of the inclusive branching fraction of $${b} $$-hadrons to an $$\eta _c (1S)$$ meson. It is consistent with a previous 90% confidence level upper limit restricted to $${{B}} ^-$$ and $${\overline{{B}}{}} {}^0$$ decays, $${\mathcal{B}}( {{{B}} ^-}, {{\overline{{B}}{}} {}^0} {\rightarrow } \eta _c (1S) X ) < 9 \times 10^{-3}$$ [15].

The prompt production yield ratios at the different centre-of-mass energies are obtained as

$$\begin{aligned} ( N^{P}_{\eta _c (1S)}/N^{P}_{{{{ J}}/{\psi }}} )_{{\sqrt{s}} = 7{~\mathrm{TeV}}}&= 1.24 \pm 0.21 \pm 0.20, \\ ( N^{P}_{\eta _c (1S)}/N^{P}_{{{{ J}}/{\psi }}} )_{{\sqrt{s}} = 8{~\mathrm{TeV}}}&= 1.14 \pm 0.21 \pm 0.18. \end{aligned}$$

After correcting with the ratio of branching fractions ${\mathcal{B}}( {{{ J}}/{\psi }} {\rightarrow } {{{ p}}} \overline{{{{ p}}}}) / {\mathcal{B}}( \eta _c (1S) {\rightarrow } {{{ p}}} \overline{{{{ p}}}})$ [21], the relative $\eta _c (1S)$ to ${{ J}}/{\psi }$ prompt production rates in the kinematic regime $2.0 < y < 4.5$ and $p_\mathrm{T} > 6.5 \mathrm{{\,GeV/}{ c}}$ are found to be

$$\begin{aligned} ( \sigma _{\eta _c (1S)} / \sigma _{{{{ J}}/{\psi }}} )_{{\sqrt{s}} = 7{~\mathrm{TeV}}} = 1.74 \pm 0.29 \pm 0.28 \pm 0.18 _{{\mathcal{B}}}, \end{aligned}$$

for the data sample collected at ${\sqrt{s}} = 7{~\mathrm{TeV}}$, and

$$\begin{aligned} ( \sigma _{\eta _c (1S)} / \sigma _{{{{ J}}/{\psi }}} )_{{\sqrt{s}} = 8{~\mathrm{TeV}}} = 1.60 \pm 0.29 \pm 0.25 \pm 0.17 _{{\mathcal{B}}} , \end{aligned}$$

for the data sample collected at ${\sqrt{s}} = 8{~\mathrm{TeV}}$. The third contribution to the uncertainty is due to that on the ${{{ J}}/{\psi }} {\rightarrow } {{{ p}}} \overline{{{{ p}}}}$ and $\eta _c (1S) {\rightarrow } {{{ p}}} \overline{{{{ p}}}}$ branching fractions.

The absolute $\eta _c (1S)$ prompt cross-section is calculated using the ${{ J}}/{\psi }$ prompt cross-section measured in Refs. [2] and [3] and integrated in the kinematic range of the present analysis, $2.0 < y < 4.5$ and $p_\mathrm{T} > 6.5 \mathrm{{\,GeV/}{ c}}$. The corresponding ${{ J}}/{\psi }$ prompt cross-sections were determined to be $( \sigma _{{{{ J}}/{\psi }}} )_{{\sqrt{s}} = 7{~\mathrm{TeV}}} = 296.9 \pm 1.8 \pm 16.9 \mathrm \,nb$ for ${\sqrt{s}} = 7{~\mathrm{TeV}}$ [2], and $( \sigma _{{{{ J}}/{\psi }}} )_{{\sqrt{s}} = 8{~\mathrm{TeV}}} = 371.4 \pm 1.4 \pm 27.1 \mathrm \,nb$ for ${\sqrt{s}} = 8 {~\mathrm{TeV}}$ [3]. The ${{ J}}/{\psi }$ meson is assumed to be produced unpolarized. The prompt $\eta _c (1S)$ cross-sections in this kinematic region are determined to be

$$\begin{aligned} ( \sigma _{\eta _c (1S)} )_{{\sqrt{s}} = 7{~\mathrm{TeV}}} = 0.52 \pm 0.09 \pm 0.08 \pm 0.06 _{\sigma _{{{{ J}}/{\psi }}} , \, {\mathcal{B}}} \mathrm {~\upmu b} , \end{aligned}$$

for $${\sqrt{s}} = 7 {~\mathrm{TeV}}$$, and

$$\begin{aligned} ( \sigma _{\eta _c (1S)} )_{{\sqrt{s}} = 8{~\mathrm{TeV}}} = 0.59 \pm 0.11 \pm 0.09 \pm 0.08 _{\sigma _{{{{ J}}/{\psi }}} , \, {\mathcal{B}}} \mathrm {~\upmu b}, \end{aligned}$$

for $${\sqrt{s}} = 8 {~\mathrm{TeV}}$$. Uncertainties associated with the $${{{ J}}/{\psi }} {\rightarrow } {{{ p}}} \overline{{{{ p}}}} $$ and $$\eta _c (1S) {\rightarrow } {{{ p}}} \overline{{{{ p}}}} $$ branching fractions, and with the $${{ J}}/{\psi } $$ cross-section measurement, are combined into the last uncertainty component, dominated by the knowledge of the branching fractions. This is the first measurement of prompt $$\eta _c (1S)$$ production in $${{ p}} $$$${{ p}} $$ collisions. The cross-section for the $$\eta _c (1S)$$ prompt production is in agreement with the colour-singlet leading order (LO) calculations, while the predicted cross-section exceeds the observed value by two orders of magnitude when the colour-octet LO contribution is taken into account [33]. However, the NLO contribution is expected to significantly modify the LO result [11]. Future measurements at the LHC design energy of $${\sqrt{s}} = 14 {~\mathrm{TeV}}$$ may allow a study of the energy dependence of the $$\eta _c (1S)$$ prompt production.

**Fig. 3:**
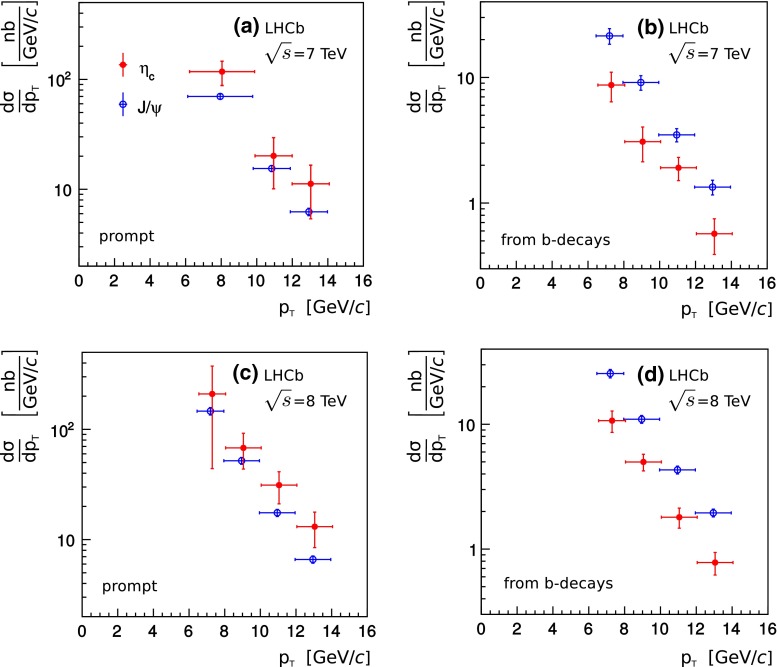
Transverse momentum spectra for $$\eta _c (1S)$$ mesons (*red filled circles*). The $$p_\mathrm{T}$$ spectra of $${{ J}}/{\psi } $$ from Refs. [1–3] are shown for comparison as *blue open circles*. Prompt production spectra are shown on **a** and **c** for data collected at $${\sqrt{s}} = 7 {~\mathrm{TeV}}$$ and $${\sqrt{s}} = 8 {~\mathrm{TeV}}$$, respectively. The spectra from inclusive charmonium production in $${b} $$-hadron decays are shown on **b** and **d** for data collected at $${\sqrt{s}} = 7 {~\mathrm{TeV}}$$ and $${\sqrt{s}} = 8 {~\mathrm{TeV}}$$, respectively

The $$\eta _c (1S)$$ differential cross-section as a function of $$p_\mathrm{T}$$ is obtained by fitting the $$p\overline{p}$$ invariant mass spectrum in three or four bins of $$p_\mathrm{T}$$. The same procedure as used to extract the $$\eta _c (1S)$$ cross-section is followed. The $${{ J}}/{\psi } $$$$p_\mathrm{T}$$ spectrum measured in Refs. [1–3] is used to obtain the $$\eta _c (1S)$$$$p_\mathrm{T}$$ spectrum for both prompt production and inclusive $$\eta _c (1S)$$ production in $${b} $$-hadron decays (Fig. 3). The $$p_\mathrm{T}$$ dependence of the $$\eta _c (1S)$$ production rate exhibits similar behaviour to the $${{ J}}/{\psi } $$ meson rate in the kinematic region studied.

The performance of the LHCb tracking system and the use of a final state common to $${{ J}}/{\psi } $$ and $$\eta _c (1S)$$ decays allows a precise measurement of the mass difference between the two mesons. In order to measure the $$\eta _c (1S)$$ mass relative to the well-reconstructed and well-known $${{ J}}/{\psi } $$ mass, a momentum scale calibration [34] is applied on data, and validated with the $${{ J}}/{\psi } $$ mass measurement. The $$M_{{{{ J}}/{\psi }}}$$ and $$\Delta M _{{{{ J}}/{\psi }}, \, \eta _c (1S)}$$ values are extracted from the fit to the $$p\overline{p}$$ invariant mass in the low-background sample of charmonium candidates produced in $${b} $$-hadron decays (Fig. 1). The $${{ J}}/{\psi } $$ mass measurement, $$M_{{{{ J}}/{\psi }}} = 3096.66 \pm 0.19 \pm 0.02$$ $${\mathrm {\,MeV\!/}c^2}$$, agrees well with the average from Ref. [21]. The mass difference is measured to be

$$\begin{aligned} \Delta M_{{{{ J}}/{\psi }}, \, \eta _c (1S)} = 114.7 \pm 1.5 \pm 0.1 \, {\mathrm {\,MeV\!/}c^2}. \end{aligned}$$

The systematic uncertainty is dominated by the parametrization of the ${{{ J}}/{\psi }} {\rightarrow } {{{ p}}} \overline{{{{ p}}}} {{\pi } ^0}$ contribution. The mass difference agrees with the average from Ref. [21]. In addition, the $\eta _c (1S)$ natural width is obtained from the fit to the $p\overline{p}$ invariant mass (Fig. 1), $\Gamma _{\eta _c (1S)} = 25.8 \pm 5.2 \pm 1.9 \mathrm {\,MeV}$. The systematic uncertainty is dominated by knowledge of the detector mass resolution. The value of $\Gamma _{\eta _c (1S)}$ obtained is in good agreement with the average from Ref. [21], but it is less precise than previous measurements.

## Summary

In summary, $$\eta _c (1S)$$ production is studied using $${{ p}} $$$${{ p}} $$ collision data corresponding to integrated luminosities of 0.7fb$$^{-1}$$ and 2.0fb$$^{-1}$$, collected at centre-of-mass energies $${\sqrt{s}} = 7 {~\mathrm{TeV}}$$ and $${\sqrt{s}} = 8 {~\mathrm{TeV}}$$, respectively. The inclusive branching fraction of $${b} $$-hadron decays into $$\eta _c (1S)$$ mesons with $$p_\mathrm{T} > 6.5 \mathrm{{\,GeV/}{ c}} $$, relative to the corresponding fraction into $${{ J}}/{\psi } $$ mesons, is measured, for the first time, to be

$$\begin{aligned} {\mathcal{B}}( {{b}} {\rightarrow } \eta _c (1S) X ) / {\mathcal{B}}( {{b}} {\rightarrow } {{{ J}}/{\psi }} X ) = 0.421 \pm 0.055 \pm 0.025 \pm 0.045 _{{\mathcal{B}}}. \end{aligned}$$

The first uncertainty is statistical, the second is systematic, and the third is due to uncertainties in the branching fractions of $$\eta _c (1S)$$ and $${{ J}}/{\psi } $$ meson decays to the $$p\overline{p}$$ final state. The inclusive branching fraction of $${b} $$-hadrons into $$\eta _c (1S)$$ mesons is derived as

$$\begin{aligned} {\mathcal{B}}( b {\rightarrow } \eta _c (1S) X ) = (4.88 \pm 0.64 \pm 0.25 \pm 0.67 _{{\mathcal{B}}}) \times 10^{-3}, \end{aligned}$$

where the third uncertainty component includes also the uncertainty on the inclusive branching fraction of $${b} $$-hadrons into $${{ J}}/{\psi } $$ mesons. The $$\eta _c (1S)$$ prompt production cross-section in the kinematic region $$2.0 < y < 4.5$$ and $$p_\mathrm{T} > 6.5 \mathrm{{\,GeV/}{ c}} $$, relative to the corresponding $${{ J}}/{\psi } $$ meson cross-section, is measured, for the first time, to be

$$\begin{aligned} ( \sigma _{\eta _c (1S)}/\sigma _{{{{ J}}/{\psi }}} )_{{\sqrt{s}} = 7{~\mathrm{TeV}}}&= 1.74 \pm 0.29 \pm 0.28 \pm 0.18 _{{\mathcal{B}}}, \\ ( \sigma _{\eta _c (1S)}/\sigma _{{{{ J}}/{\psi }}} )_{{\sqrt{s}} = 8{~\mathrm{TeV}}}&= 1.60 \pm 0.29 \pm 0.25 \pm 0.17 _{{\mathcal{B}}}, \end{aligned}$$

where the third uncertainty component is due to uncertainties in the branching fractions of $\eta _c (1S)$ and ${{ J}}/{\psi }$ meson decays to the $p\overline{p}$ final state. From these measurements, absolute $\eta _c (1S)$ prompt cross-sections are derived, yielding

$$\begin{aligned} ( \sigma _{\eta _c (1S)} )_{{\sqrt{s}} = 7{~\mathrm{TeV}}}&= 0.52 \pm 0.09 \pm 0.08 \pm 0.06 _{\sigma _{{{{ J}}/{\psi }}} , \, {\mathcal{B}}} \mathrm {~\upmu b}, \\ ( \sigma _{\eta _c (1S)} )_{{\sqrt{s}} = 8{~\mathrm{TeV}}}&= 0.59 \pm 0.11 \pm 0.09 \pm 0.08 _{\sigma _{{{{ J}}/{\psi }}} , \, {\mathcal{B}}} \mathrm {~\upmu b}, \end{aligned}$$

where the third uncertainty includes an additional contribution from the ${{ J}}/{\psi }$ meson cross-section. The above results assume that the ${{ J}}/{\psi }$ is unpolarized. The $\eta _c (1S)$ prompt cross-section is in agreement with the colour-singlet LO calculations, whereas the colour-octet LO contribution predicts a cross-section that exceeds the observed value by two orders of magnitude [33]. The $p_\mathrm{T}$ dependences of the $\eta _c (1S)$ and ${{ J}}/{\psi }$ production rates exhibit similar behaviour in the kinematic region studied. The difference between the ${{ J}}/{\psi }$ and $\eta _c (1S)$ meson masses is also measured, yielding $\Delta M_{{{{ J}}/{\psi }}, \, \eta _c (1S)} = 114.7 \pm 1.5 \pm 0.1 {\mathrm {\,MeV\!/}c^2}$, where the first uncertainty is statistical and the second is systematic. The result is consistent with the average from Ref. [21].
